# Cavitary lung squamous cell carcinoma misdiagnosed as pulmonary aspergillosis following targeted next-generation sequencing: A case report

**DOI:** 10.1097/MD.0000000000051432

**Published:** 2026-10-30

**Authors:** Ya Shen, Xiao-Yu Cao, Jing-Feng Shi, Xiao-Bao Teng, Ming-Feng Han

**Affiliations:** a Department of Respiratory and Critical Care Medicine, Fuyang Infectious Disease Clinical College of Anhui Medical University, Fuyang, Anhui, China.

**Keywords:** aspergillosis, case report, cavitary lung cancer, endobronchial ultrasound-guided transbronchial needle aspiration, squamous cell carcinoma, targeted next-generation sequencing

## Abstract

**Rationale:**

Cavitary lung cancer, particularly squamous cell carcinoma (SCC), can closely mimic infectious cavitary diseases such as pulmonary aspergillosis. When molecular pathogen testing yields a positive result, the infectious diagnosis may be erroneously reinforced, and histological confirmation may be delayed.

**Patient concerns:**

A 68-year-old male farmer with a 40 pack-year smoking history presented with a 6-month history of cough, expectoration, and chest tightness. Chest computed tomography revealed a 66 mm × 70 mm thick-walled cavitary lesion in the left upper lobe. Targeted next-generation sequencing of bronchoalveolar lavage fluid at the referring hospital detected *Aspergillus flavus* sequences, leading to a misdiagnosis of pulmonary aspergillosis and 2.5 months of voriconazole therapy, during which the lesion progressed.

**Diagnoses:**

Repeat bronchoscopy at our institution revealed a fistulous opening with intracavitary nodules and necrotic material. Endobronchial ultrasound-guided transbronchial needle aspiration (EBUS-TBNA) at stations 4 L and 10 L confirmed poorly differentiated lung SCC. 18F-fluorodeoxyglucose positron emission tomography/computed tomography (PET-CT) demonstrated hypermetabolism in the primary lesion, mediastinal lymph nodes, and sternum, supporting a clinical-radiological diagnosis of stage IVA (cT4N2M1b) disease.

**Interventions:**

The patient received first-line paclitaxel plus carboplatin combined with tislelizumab for 6 cycles, followed by tislelizumab maintenance monotherapy.

**Outcomes:**

A partial response according to the Response Evaluation Criteria in Solid Tumors version 1.1 was achieved after the first cycle, with sustained tumor shrinkage after 6 cycles and no evidence of disease progression during ongoing maintenance therapy.

**Lessons:**

In cavitary lung lesions, targeted next-generation sequencing detection of fungal sequences should not by itself override the need for histological evaluation, because molecular assays cannot distinguish colonization or contamination from invasive infection. Endobronchial ultrasound-guided transbronchial needle aspiration combined with positron emission tomography/computed tomography is valuable for diagnosis and staging when cavitary malignancy is suspected, and SCC remains an important subtype of cavitary lung cancer in long-term smokers.

## 1. Introduction

Lung cancer remains the leading cause of cancer-related mortality worldwide.^[1,2]^ Cavitation within the tumor is a well-recognized radiological phenomenon; up to 16% of lung cancers exhibit cavitation at diagnosis, most commonly in squamous cell carcinoma (SCC), in which rapid growth outpacing blood supply leads to central necrosis and liquefaction.^[3]^ Cavitary lung cancer of this necrotic, thick-walled type should be distinguished from lung cancer associated with cystic airspaces, a separate radiological category characterized by thin-walled cystic or bullous spaces within or adjacent to the tumor and a predominance of adenocarcinoma histology.^[4]^ The present case concerns the former category, that is, a thick-walled cavitary SCC.

The radiological overlap between cavitary lung cancer and infectious cavitary diseases, particularly chronic pulmonary aspergillosis and aspergilloma, poses a considerable diagnostic challenge. Both entities may present as thick-walled cavitary lesions with intracavitary soft tissue components, and coexisting chronic lung disease or immunosuppression can further complicate the clinical picture. Notably, up to 16% of lung cancers exhibit cavitation at diagnosis, and central necrosis resulting from rapid tumor growth that outpaces blood supply is particularly common in SCC.^[4]^

In recent years, targeted next-generation sequencing (tNGS) and metagenomic next-generation sequencing (mNGS) have become powerful tools for pathogen detection in pulmonary infections, offering high sensitivity and rapid turnaround times.^[5,6]^ However, these molecular techniques cannot reliably differentiate active infection from airway colonization or environmental contamination, particularly in elderly patients, chronic smokers, and individuals with structural lung disease. Among these, Aspergillus colonization is well documented.^[6]^

A positive molecular result may therefore induce diagnostic anchoring bias, delaying oncological diagnosis. Reports in which a positive tNGS result itself anchored the misdiagnosis of cavitary lung SCC as pulmonary aspergillosis in an immunocompetent patient remain scarce.

Here, we report such a case, in which detection of 2762 *A flavus* reads in bronchoalveolar lavage fluid (BALF) led to more than 2 months of unnecessary antifungal therapy before repeat bronchoscopy with endobronchial ultrasound-guided transbronchial needle aspiration (EBUS-TBNA) and positron emission tomography/computed tomography (PET-CT) established the correct diagnosis and stage. We describe the complete diagnostic journey, with serial imaging, pathological confirmation, staging, and the subsequent response to chemoimmunotherapy, and we discuss how molecular microbiological results should be weighed within the diagnostic evidence chain for cavitary lung lesions.

## 2. Case presentation

A 68-year-old male farmer developed a cough, expectoration, and chest tightness in February 2025. He had a 40 pack-year smoking history and denied any history of occupational dust exposure, prior tuberculosis, immunosuppressive therapy, or a family history of malignancy. The cough was productive with yellow-white sputum that was occasionally brick-red, accompanied by chest tightness and dyspnea, without fever or chest pain. Physical examination on admission was largely unremarkable, except for locally decreased breath sounds over the left upper lung field, and vital signs were within normal limits.

On August 14, 2025, chest computed tomography (CT) performed at a local hospital (Jieshou People’s Hospital) revealed a cavitary lesion in the left upper lobe adjacent to the hilum, measuring approximately 66 mm × 70 mm, with clear margins, uneven wall thickness (maximum wall thickness was 16 mm), and thickening of the adjacent bronchial wall (Fig. 1A). Bronchoscopy was performed on August 20, 2025. Three biopsy specimens obtained from the cavity wall and perilesional bronchial mucosa showed necrotic debris and chronic inflammatory tissue without malignant cells. Targeted next-generation sequencing tNGS of BALF, performed on the same occasion, detected 2762 sequence reads of *Aspergillus flavus, 3447* reads of *human herpesvirus type 5,* and 732 reads of *human herpesvirus type 6B*. tNGS was performed with a commercial 337-pathogen respiratory panel (Anweikang Diagnostics, Hefei, China). After specimen liquefaction, mechanical lysis, and nucleic acid extraction, libraries underwent multiplex PCR-based target enrichment and single-end 100-bp sequencing; reads were quality-filtered, host-subtracted against the human reference genome, and mapped to a curated pathogen database. Positivity thresholds followed the Chinese expert consensus, at least 15 reads per hundred thousand for bacteria.^[7]^ Serum galactomannan and (1–3)-β-D-glucan assays were negative. A diagnosis of pulmonary aspergillosis was made, and oral voriconazole (dose, 200 mg twice daily) was initiated on August 23, 2025.

**Figure 1.**
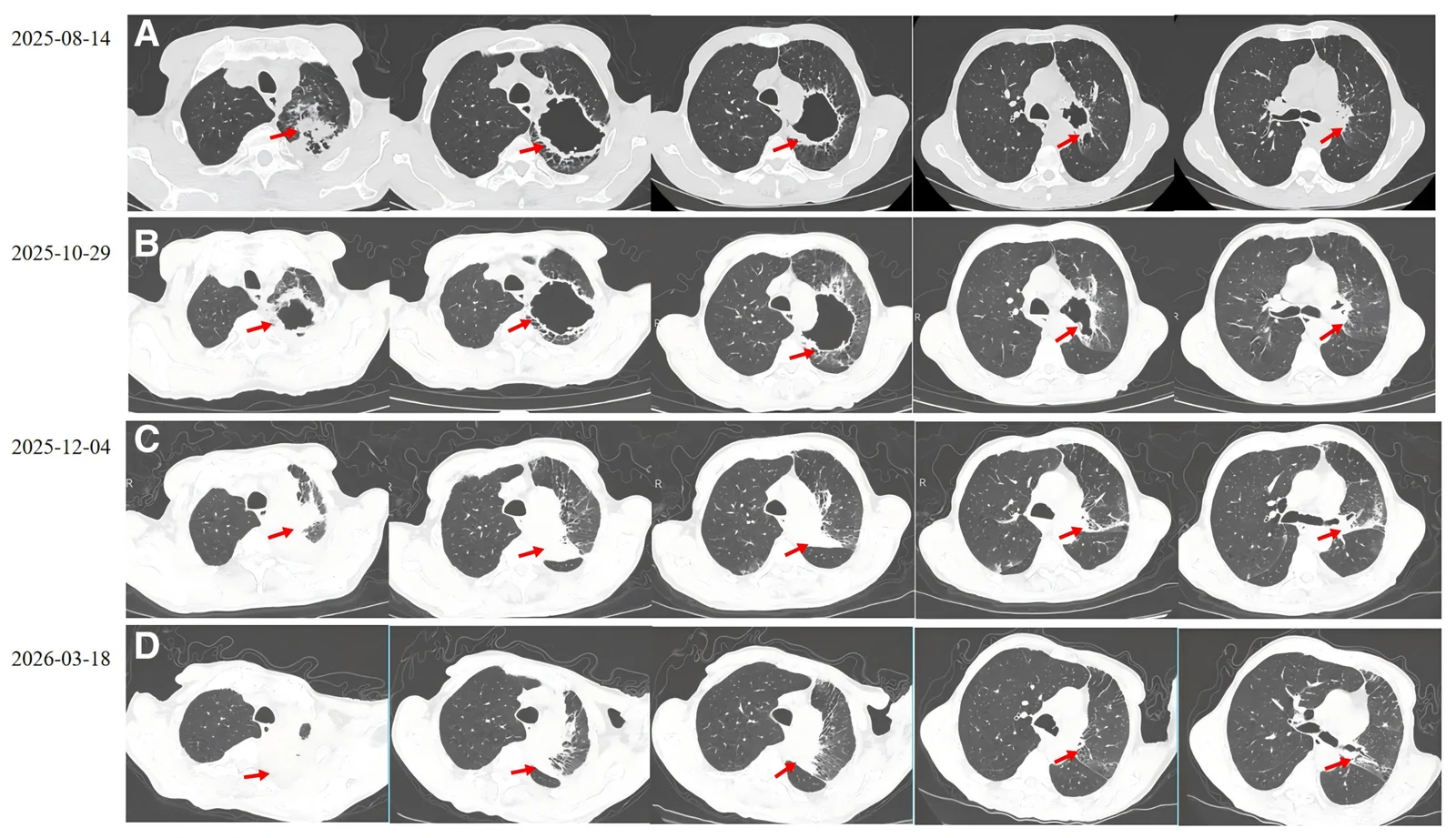
Serial chest CT images showing the evolution of the cavitary lesion. Similar anatomical levels were selected for each row. Arrows indicate the cavitary wall and mural nodules. (A) August 14, 2025: thick-walled cavitary lesion in the left upper lobe at the referring hospital. (B) October 29, 2025: lesion progression after 2 months of antifungal therapy. (C) December 4, 2025: tumor shrinkage after 1 cycle of paclitaxel, carboplatin, and tislelizumab. (D) March 18, 2026: continued regression after 6 cycles. CT = computed tomography.

On October 29, 2025, follow-up chest CT demonstrated lesion progression compared with the previous scan (Figure 1B, longest diameter 76 mm). Because cough and chest tightness persisted, the patient was referred to our hospital for further evaluation. The interval between the first presentation and referral to a tertiary center was therefore approximately 11 weeks.

The patient was admitted to the Department of Respiratory and Critical Care Medicine at our institution on October 31, 2025. Repeat bronchoscopy on November 4, 2025 revealed a fistulous opening in the apical segment of the left upper lobe (Fig.2A); narrow-band imaging showed abnormal mucosal vascular patterns (Fig.2B); and exploration of the distal cavity revealed irregular intracavitary nodules and black necrotic material (Fig.2C). Endobronchial ultrasound identified enlarged lymph nodes at stations 4L and 10L, and EBUS-TBNA was performed (Fig.2D). Histopathological and immunohistochemical examinations confirmed poorly differentiated lung SCC, and tumor cells were positive for P40, P63, and CK7 and negative for TTF-1, as shown in Figure 3. The Ki-67 proliferation index was approximately 50%, and the programmed death-ligand 1 (PD-L1) tumor proportion score was below 1% (clone E1L3N). A mycological work-up was performed in parallel at our institution after the patient was admitted to the hospital: serum galactomannan was 0.262 ug/L (reference < 0.5 ug/L), serum (1-3)-β-D-glucan was 36 pg/mL (reference < 70 pg/mL), BALF direct microscopy showed no fungal hyphae, and BALF fungal culture yielded no Aspergillus growth after 1 week.

**Figure 2.**
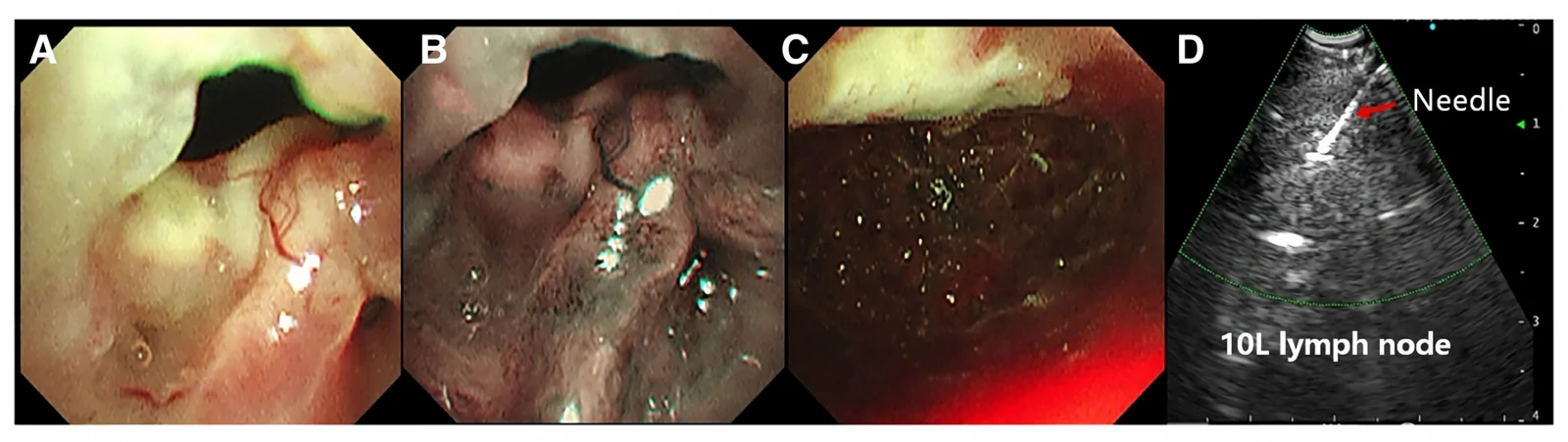
Bronchoscopic and endobronchial ultrasound images. (A) Fistulous opening in the apical segment of the left upper lobe. (B) Narrow-band imaging showing abnormal vascular patterns. (C) Intracavitary view revealing irregular nodular lesions and black necrotic material. (D) Endobronchial ultrasound-guided transbronchial needle aspiration of the station 10L lymph node. The lymph node and puncture needle position are marked.

**Figure 3.**
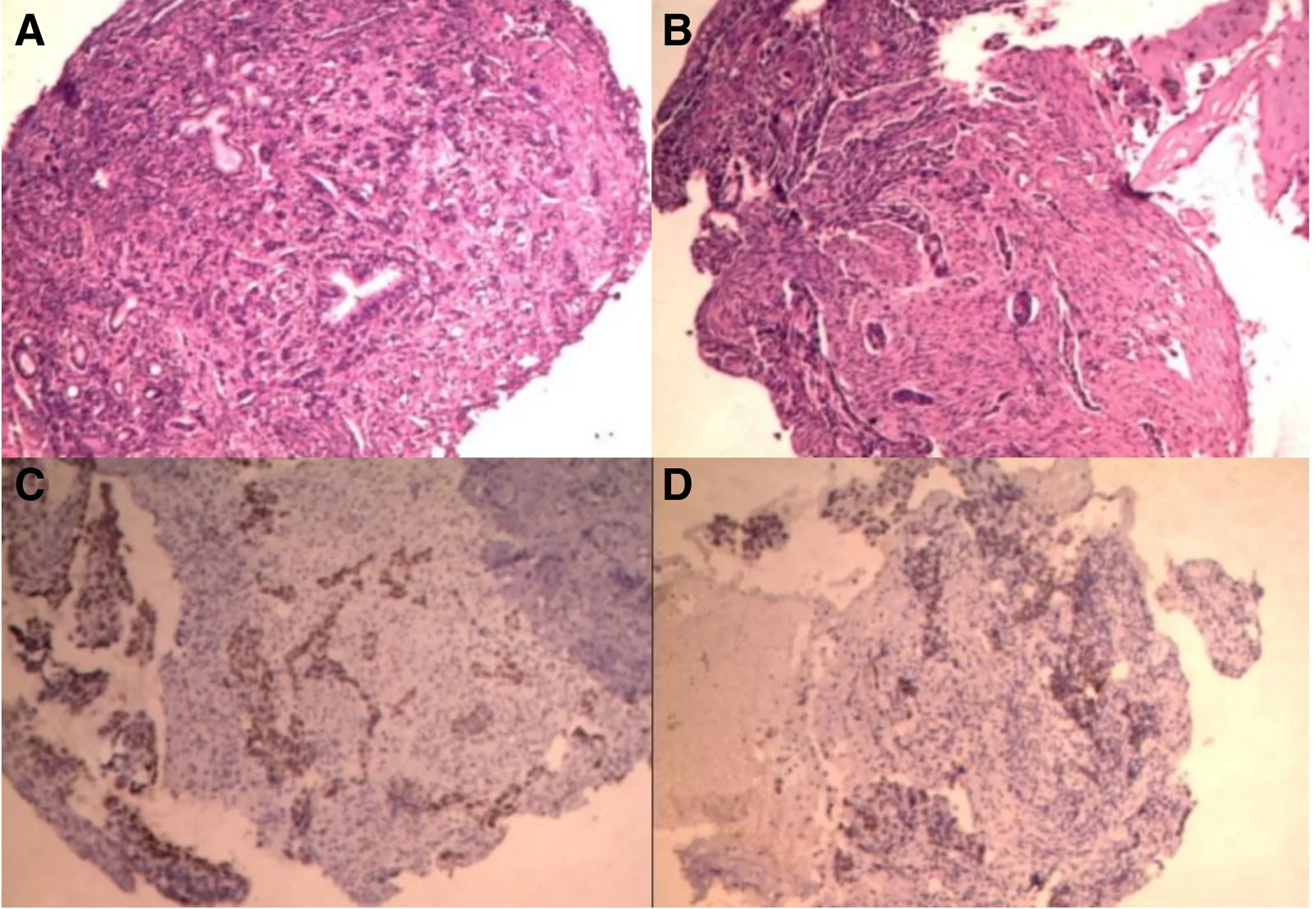
Histopathological and immunohistochemical images of EBUS-TBNA specimens from mediastinal lymph nodes. (A) Hematoxylin and eosin (HE) staining at 100x magnification. (B) HE staining at 100x magnification showing poorly differentiated squamous cell carcinoma. (C) P40 immunohistochemical staining at 100x magnification. Tumor cells show positive expression. (D) P63 immunohistochemical staining at 100x magnification. Tumor cells show positive expression.

18F-fluorodeoxyglucose (FDG) positron emission tomography/computed tomography (PET-CT) performed on November 7, 2025 showed intense hypermetabolism in the left upper lobe lesion maximum standardized uptake value (SUVmax), 14.7 and mediastinal lymph nodes (SUVmax, 10.3), and a focal area of increased uptake in the sternum (SUVmax, 7.5) with a corresponding osteolytic change on the CT component. Subsequent chest bone magnetic resonance imaging examinations also considered being metastatic tumor foci. The sternal lesion was therefore classified as a solitary extrathoracic metastasis on clinical-radiological grounds rather than by pathological confirmation. According to the eighth edition of the American Joint Committee on Cancer staging system,^[8]^ the tumor was classified as cT4 (greatest dimension 7.0 cm, exceeding the 7-cm threshold), cN2 (metastasis to ipsilateral mediastinal lymph node station 4L), and cM1b (a single extrathoracic metastatic lesion), corresponding to clinical stage IVA.

On November 11, 2025, the patient commenced first-line systemic therapy with paclitaxel (175 mg/m^2^, day 1), carboplatin (area under the curve 5, day 1), and the anti-programmed death-1 antibody tislelizumab (200 mg, day 1), administered once every 3 weeks for a planned 6 cycles. The treatment course was well tolerated, with no dose reductions, treatment delays, or grade ≥ 3 adverse events/ details of any adjustments.

Tumor response was assessed according to the Response Evaluation Criteria in Solid Tumors version 1.1 (RECIST 1.1).^[9]^ The left upper lobe cavitary mass was selected as the target lesion; its longest diameter was measured on axial CT images at consistent window settings, with the cavity included within the outer tumor margin (that is, the overall lesion diameter was measured rather than the solid component alone). The pretreatment scan of October 29, 2025, served as the RECIST baseline. Chest CT on December 4, 2025, after the first treatment cycle, showed marked shrinkage of the target lesion to 63 mm, corresponding to a 17% decrease from baseline and meeting the criteria for a partial response (Fig.1C). The patient completed 6 cycles of the same regimen, and follow-up CT on March 18, 2026, demonstrated continued regression of the primary lesion to 45 mm, a 40% decrease from baseline, consistent with sustained partial response (Fig.1D).

The patient is currently receiving tislelizumab maintenance monotherapy 200 mg every 3 weeks, planned for up to 2 years in the absence of progression or unacceptable toxicity. Surveillance comprises clinical assessment and contrast-enhanced chest CT every 2 cycles (approximately 6–9 weeks), together with laboratory monitoring including blood counts, hepatic and renal function, and thyroid function for immune-related adverse events. At the most recent assessment on June 28, 2026, there was no evidence of disease progression; the next evaluation is scheduled for September 2026. Key diagnostic and therapeutic events are summarized in the timeline shown in Figure 4.

**Figure 4.**
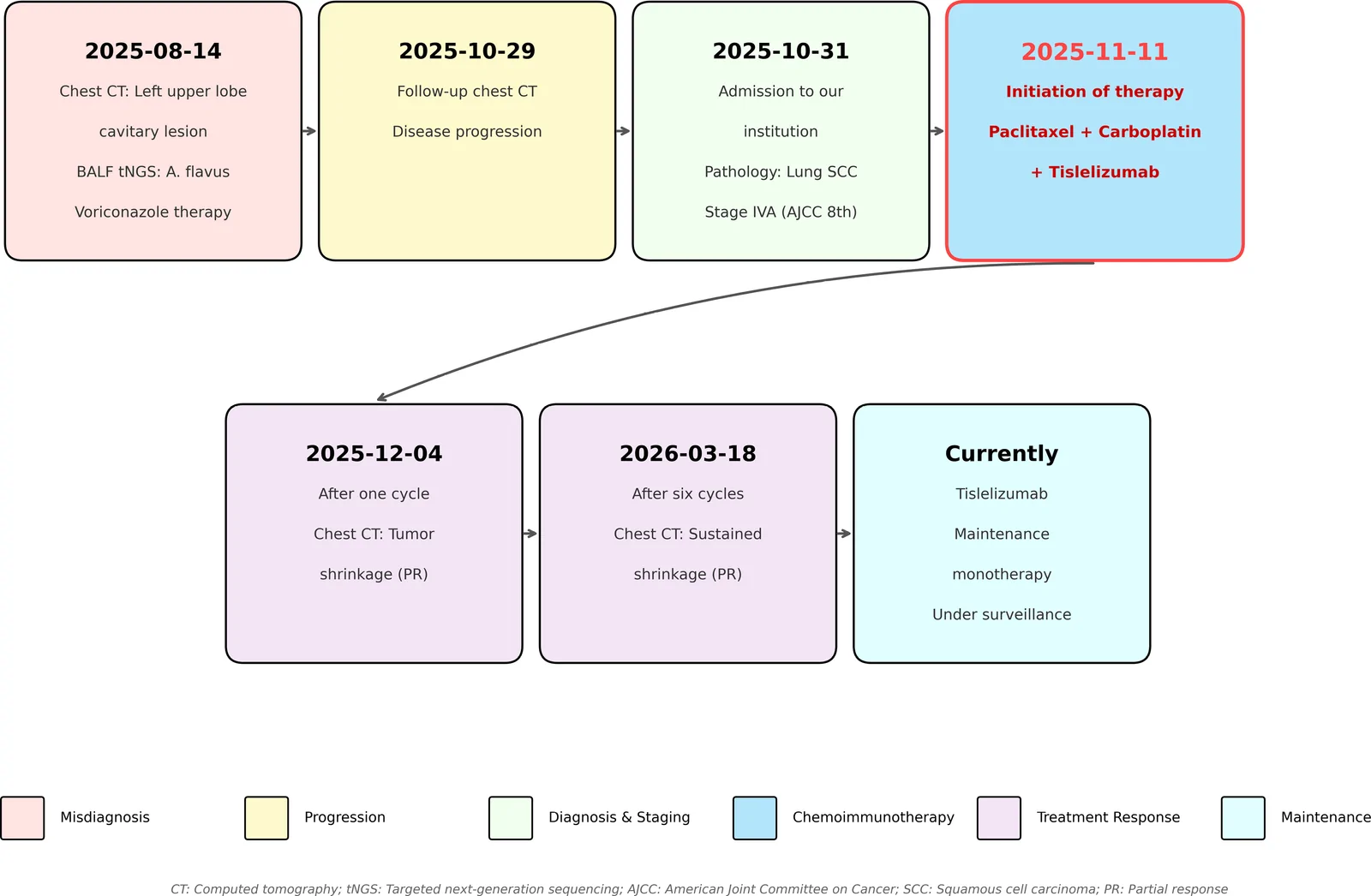
Timeline of key diagnostic and therapeutic events. The patient initially presented to a county-level hospital with a thick-walled cavitary lesion. Targeted next-generation sequencing (tNGS) detected *Aspergillus flavus* sequences in bronchoalveolar lavage fluid (BALF), leading to a diagnosis of pulmonary aspergillosis and approximately 2.5 months of voriconazole therapy. Following disease progression, endobronchial ultrasound-guided transbronchial needle aspiration (EBUS-TBNA) confirmed lung squamous cell carcinoma (SCC), and positron emission tomography/computed tomography (PET-CT) established stage IVA disease. First-line chemoimmunotherapy with paclitaxel, carboplatin, and tislelizumab achieved a rapid partial response (PR) after 1 cycle, with sustained tumor shrinkage after six cycles. The patient is currently receiving tislelizumab maintenance monotherapy.BALF = bronchoalveolar lavage fluid, CT = computed tomography, positron emission tomography/computed tomography, PET-CT = positron emission tomography/computed tomography, SCC = squamous cell carcinoma, tNGS = targeted next-generation sequencing.

When informed of the final diagnosis, the patient expressed initial frustration regarding the earlier antifungal treatment; however, a definitive diagnosis was established. He reported a substantial improvement in cough and chest tightness after the first treatment cycle and stated that he felt well supported throughout his care. He hopes that sharing his experience will help other patients with similar lesions receive a timely diagnosis.

## 3. Discussion

Differential diagnosis of cavitary lung lesions remains one of the most challenging scenarios in thoracic imaging. Malignant cavities, particularly those arising from SCC, typically exhibit thick (>8 mm), irregular walls with nodular inner margins, eccentrically located soft tissue, and neovascularization on contrast-enhanced CT.^[10]^ In contrast, aspergillomas classically present as mobile, rounded intracavitary masses within preexisting thin-walled cavities, often demonstrating the air-crescent (Monod) sign with an upper lobe predominance.^[11]^ Chronic pulmonary aspergillosis may also manifest as thick-walled cavities, pleural thickening, and adjacent parenchymal infiltrates, features that overlap considerably with cavitary malignancy.

The first CT scan in this patient revealed a thick-walled cavity. The wall measured up to 16 mm at its thickest point; the inner surface appeared uneven, and soft-tissue elements sat off-center rather than layering dependently. In addition, it was observed that there was an increase in the thickness of the bronchial walls in the adjacent areas. Reviewing these examination results suggests the possibility of malignant lesions. Park et al^[10]^ demonstrated that malignant cavities exhibit significantly greater wall thickness and more pronounced mural nodule enhancement than aspergillomas and that mural nodules in lung cancer typically occupy nondependent positions within the cavity. Lal et al^[12]^ further reported that multidetector CT features, such as wall thickness >8 mm, irregular inner margins, and lobulated outer margins, are independent predictors of malignancy in cavitary lesions, with a diagnostic accuracy exceeding 85%. However, we are not suggesting that the initial CT scan alone could have reliably separated these 2 conditions. Instead, we view the imaging findings as warning signs that ought to have triggered early tissue sampling alongside microbiological workup, not after it or instead of it. The fact that these warning signs were overlooked during the first reading of the scan played a major part in prolonging the time to diagnosis.

The integration of tNGS into clinical microbiology workflows has undoubtedly improved pathogen detection rates, particularly for fastidious and slow-growing organisms. Nevertheless, our case illustrates the importance of interpreting molecular results within the full clinical context. tNGS detects microbial nucleic acids but cannot distinguish active infection from airway colonization or environmental contamination. Aspergillus species are ubiquitous in the environment, and airway colonization is common in elderly patients, smokers, and individuals with chronic lung diseases.

In our case, the absence of serum galactomannan positivity, negative BALF culture, and lack of radiological response to 2.5 months of voriconazole collectively argued against true invasive aspergillosis. However, based on this evidence, we cannot completely rule out chronic pulmonary aspergillosis. This is because there are several potential factors to consider: the problem of drug penetration into the cavitated lesions, the possible azole drug resistance of *A flavus*, and the relatively slow treatment response of chronic aspergillosis itself. On balance, the tNGS finding is most consistent with airway colonization or contamination of limited clinical significance rather than invasive infection, although a degree of residual uncertainty remains. The wrong diagnosis in this instance came less from a faulty test and more from cognitive anchoring. Once the molecular result returned positive, it was allowed to dominate clinical reasoning while imaging findings that did not match were pushed to the background. The subsequent failure of empirical treatment was also overlooked. Each of these events was a warning signal that should have caused the team to question the infectious explanation and go back for tissue proof.

Several studies have revealed that positive NGS test results for Aspergillus may occur in lung cancer patients. Tang et al^[13]^ showed that both mNGS and tNGS frequently detect Aspergillus and other environmental fungi in BALF samples from patients ultimately diagnosed with lung cancer, without a correlation between sequence counts and histopathological evidence of infection. A 2026 comprehensive review by Rong et al^[14]^ emphasized that NGS technologies remain largely qualitative, cannot differentiate colonization from infection, and must be integrated with clinical, radiological, and conventional microbiological data. A positive molecular result may, therefore, induce diagnostic anchoring bias, delaying oncological diagnosis. Conversely, host-derived sequencing data can occasionally point toward malignancy, as illustrated by a report of lung SCC diagnosed through copy-number variation analysis of mNGS data.^[15]^

Misdiagnosis between cavitary lung cancer and pulmonary aspergillosis has been reported in both directions. Bu et al^[16]^ described invasive pulmonary aspergillosis misdiagnosed as lung cancer, whereas Yu et al^[17]^ reported lung SCC mimicking recurrent aspergillosis in a patient with systemic lupus erythematosus, in whom anchoring on the prior infectious diagnosis delayed recognition of malignancy. Chaudhary et al^[18]^ described SCC misdiagnosed as cryptogenic organizing pneumonia. We are aware of very few prior reports like this one. A positive BALF tNGS result led clinicians to label cavitary lung SCC as an infection in an immunocompetent patient. The assay showed a high read count for A. flavus. That molecular finding alone anchored the wrong diagnosis. We also believe this is the first account that follows the entire clinical journey. It starts with the tNGS misdiagnosis. It then moves to correction by EBUS-TBNA. PET-CT staging came next. Finally, the patient showed a measurable response by RECIST 1.1 after first-line chemoimmunotherapy with tislelizumab. This case additionally underscores that SCC, rather than adenocarcinoma, should be actively considered in thick-walled necrotic cavities, complementing the literature on lung cancer associated with cystic airspaces, in which adenocarcinoma predominates.^[3]^ Looking forward, consensus reporting standards for fungal NGS (including read counts, thresholds, and control metrics), interpretive algorithms specific to cavitary lesions, and prospective studies of diagnostic pathways that mandate tissue sampling when imaging red flags or treatment failure emerge would help reduce such errors.

We do not propose that all patients with cavitary lung lesions should routinely undergo PET-CT or EBUS-TBNA. Such a recommendation would disregard the availability and cost of these modalities, particularly in resource-limited settings such as the county-level hospital at which our patient first presented. Instead, we suggest a stepwise approach. Management rests on 3 core principles. CT images should undergo careful structured reassessment for features that raise concern for malignancy, with particular attention to airway walls thicker than 8 mm, mucosal surfaces that appear irregular or nodular, soft tissue that is eccentric rather than dependent, and any thickening of the bronchial wall. When microbiological findings and imaging point in different directions, or when empirical antibiotics fail to produce a response, clinicians should move promptly to obtain new tissue. The choice of repeat bronchoscopic biopsy, endobronchial ultrasound-guided transbronchial needle aspiration, or percutaneous needle biopsy should be guided by what is technically available locally. Finally, PET-CT is best reserved for staging once histology has confirmed cancer, or for clarifying the diagnosis when initial biopsy results remain equivocal. Equally important is a low threshold for referral to a tertiary center when the diagnosis remains uncertain. In our patient, the approximately 11-week interval between first presentation and referral accounted for most of the diagnostic delay.

Once malignancy is suspected, establishing a tissue diagnosis and accurate staging becomes paramount. Our first bronchoscopic biopsy targeted the cavity wall but returned mainly inflammatory tissue with no malignant cells seen. This outcome highlights a familiar trap when sampling cavitary tumors. Because extensive necrosis often fills these lesions, the biopsy may easily miss the actual tumor. Rather than treating a negative result as definitive proof of benign disease, the clinician should actively pursue alternative sampling methods. EBUS-TBNA has emerged as the first-line minimally invasive modality for mediastinal lymph node sampling in non-small cell lung cancer (NSCLC), with pooled sensitivity and specificity of approximately 90% and 99%, respectively.^[19]^ A 2025 comprehensive review by Fiore et al^[20]^ highlighted that EBUS-TBNA not only facilitates diagnosis but also enables simultaneous mediastinal staging, thereby reducing the need for surgical mediastinoscopy. In our patient, EBUS-TBNA of stations 4L and 10L yielded sufficient tissue for histopathological diagnosis, immunohistochemical subtyping, and programmed death-ligand 1assessment, which directly guided systemic therapy.

Following pathological confirmation, 18F-FDG PET/CT played a decisive role in the staging. PET-CT is superior to conventional CT for detecting nodal metastases and distant bone involvement and can alter the stage in approximately 20% of patients. The intense FDG avidity of the primary lesion, mediastinal nodes, and the solitary sternal focus supported clinical stage IVA (cT4N2M1b) disease, excluding surgical candidacy and directing the patient toward systemic therapy. As noted above, the sternal lesion was not pathologically confirmed, and the M1b designation should be understood as a clinical-radiological diagnosis.

A 2025 meta-analysis by Yu and Chen^[21]^ confirmed that PET-CT remains the standard of care for the initial staging of NSCLC, particularly in advanced disease, where the detection of occult metastases changes management.

Immune checkpoint inhibitors combined with platinum-based chemotherapy constitute the established first-line standard of care for advanced squamous NSCLC without actionable driver mutations. The phase 3 RATIONALE-307 trial demonstrated that tislelizumab plus paclitaxel and carboplatin significantly improved progression-free survival compared with chemotherapy alone in advanced squamous NSCLC, with benefits observed irrespective of programmed death-ligand 1expression.^[22,23]^ Despite having PD-L1-negative disease, our patient achieved a rapid partial response after 1 cycle and sustained tumor shrinkage after 6 cycles, consistent with the RATIONALE-307 findings. Tumor mutational burden and tumor genomic profiling were not assessed in this patient; therefore, although high tumor mutational burden and smoking-related neoantigens are characteristic of squamous histology and have been proposed to sensitize such tumors to immunotherapy, any mechanistic attribution in this individual patient remains speculative and should be regarded only as a possible, literature-based explanation rather than a demonstrated mechanism.

This case report had several strengths. It documents the complete diagnostic course from misdiagnosis to definitive staging with serial imaging, pathology, and molecular data, and provides a real-world illustration of the limitations of tNGS in cavitary lung lesions. This study had several limitations. First, as this was a single case report, the findings cannot be generalized. Second, the sternal lesion determining M1b staging was not biopsied and is a clinical-radiological rather than pathologically confirmed metastasis. Third, the follow-up duration remained relatively short, and long-term survival outcomes have not yet been assessed.

This case highlights 3 teaching points. First, tNGS detection of fungal sequences in cavitary lung lesions should be interpreted with caution in elderly smokers whose imaging shows malignant features, and a positive molecular result should not, in isolation, override the need for histological evaluation. Second, when biopsy of the primary lesion is inconclusive, EBUS-TBNA offers a safe and minimally invasive means of obtaining diagnostic tissue from mediastinal lymph nodes. Third, SCC remains an important histological subtype of cavitary lung cancer and should not be overlooked on the assumption that cavitary or cystic lung cancers are predominantly adenocarcinomas.

## 4. Conclusion

This report describes the case of a patient with cavitary lung SCC who was misdiagnosed with pulmonary aspergillosis because tNGS detected A. flavus sequences in BALF, resulting in more than 2 months of unnecessary antifungal therapy. Repeat bronchoscopy with EBUS-TBNA was used to establish the pathological diagnosis, and PET-CT was used to stage the disease as IVA. Six cycles of paclitaxel, carboplatin, and tislelizumab achieved sustained tumor regression, and the patient continued tislelizumab maintenance.

In cavitary lung lesions, a positive molecular microbiological result does not exclude malignancy. Clinicians gain meaningful diagnostic leverage from repeat tissue sampling and EBUS-TBNA. PET-CT also holds value, though it belongs in the workup only once pathology has confirmed the diagnosis. In patients with a heavy smoking history who present with cavitary lesions, squamous cell carcinoma should remain high on the differential. We present this case mainly to underscore practical pitfalls and sensible clinical strategies. It was never intended to lay down broad diagnostic laws that apply to every patient.

## Author contributions

**Conceptualization:** Ya Shen, Ming-Feng Han.

**Data curation:** Ya Shen, Xiao-Yu Cao, Jing-Feng Shi, Xiao-Bao Teng.

**Investigation:** Ya Shen, Xiao-Yu Cao, Jing-Feng Shi, Xiao-Bao Teng.

**Supervision:** Ming-Feng Han.

**Writing – original draft:** Ya Shen, Xiao-Yu Cao, Jing-Feng Shi.

**Writing – review & editing:** Xiao-Bao Teng, Ming-Feng Han.
